# Evaluation of the* Candida* sp. 99-125 Lipase Positional Selectivity for 1,3-Diolein Synthesis

**DOI:** 10.1155/2019/4318631

**Published:** 2019-05-20

**Authors:** Yanhong Bi, Zhangqun Duan, Wenjing Zhang, Lei Xu, Zhaoyu Wang, Xiaojuan Zhao, Xiangjie Zhao, Jiali Yang

**Affiliations:** ^1^School of Life Science and Food Engineering, Huaiyin Institute of Technology, Huai'an 223003, China; ^2^Academy of State Administration of Grain, Beijing 100037, China; ^3^Faculty of Chemical Engineering, Huaiyin Institute of Technology, Huai'an 223003, China; ^4^Jiangsu Key Laboratory of Regional Resource Exploitation and Medicinal Research, Huai'an 223003, China

## Abstract

In this study, comparative experiments were carried out to investigate the positional selectivity of* Candida* sp. 99–125 lipase in preparing 1,3-diolein by using medium engineering strategy. The results indicated that the diolein yield was markedly enhanced from 56.5% to 86.7% with increasing log⁡*P* values of the solvents, while the selectivity of the examined lipase for the* sn-1* over the* sn-2* hydroxyl of glycerol was decreased, thus leading to a reduced 1,3-diolein to 1,2-diolein ratio. To evaluate the possibility of industrial enzymatic production of 1,3-diolein, larger-scale experiments were assessed. After being used repeatedly for eight batches, the diolein content reached 95.1%, while the 1,3-diolein to 1,2-diolein ratio was 7:1 following purification. Results of the* kg* level experiments significantly demonstrated the practicability of the enzymatic process and the efficiency of the purification strategy for the product.

## 1. Introduction

1,3-Diacylglycerol has positive impacts on lowering the body-fat buildup and eluding an increase in weight [1, 2]. The synthesis of 1,3-diacylglycerol via enzyme approaches has garnered greater attention, due to their environmental friendliness, safety, exquisite selectivity, and mild reaction conditions [3–6]. Meanwhile, 1,2-diacylglycerol, which is a positional isomer, can be produced at the same time. Thus, the positional selectivity of the enzyme utilized becomes pivotal to attain the elevated 1,3-diacylglycerol yield.

As the reaction media have a significant impact on selectivity of an enzyme, numerous papers have documented the impact of the physicochemical characteristics of the solvent on the selectivity of the utilized enzyme [7–9]. It has been suggested that log⁡*P*, which is the logarithm of the partition coefficient of a solvent in the standard octanol-water two-phase system, was more practical [10–12].

Further, the documented lipases (e.g., Novozyme 435 and Lipozyme RM IM) for 1,3-diacylglycerol generation are costly. A commercially available cheap lipase from* Candida *sp. 99–125 has been proved to be quite effective to catalyze preparation of fatty esters such as biodiesel, monoacylglycerol, and diacylglycerol by esterification from fatty acids [5, 13, 14]. In addition, to the best of our knowledge, the positional selectivity of* Candida* sp. 99–125 lipase in the preparation of 1,3-diacylglycerol is seldom examined. In particular, no evaluation has been noted as examining the how the positional selectivity of* Candida* sp. 99–125 lipase is influenced by the reaction media. As a result of this, we now explore the work to examine the impact of the log⁡*P* values of the solvents on the positional selectivity of* Candida* sp. 99–125 lipase, in which the model reaction for 1,3-diolein synthesis was the oleic acid esterification with glycerol. Moreover, the larger-scale experiments, operational stability of the enzyme, and purification of 1,3-diolein were also conducted to additionally evaluate the possibility of using* Candida *sp. 99–125 lipase for the industrial generation of 1,3-diolein.

## 2. Materials and Methods

### 2.1. Materials: Candida sp

99–125 lipase (lyophilized powder) was bought from Beijing CTA New Century Biotechnology Co., Ltd. The amount of protein was 370 mg protein/g enzyme powder, as determined by the Bradford assay. The standard substances of 1-monoolein, 2-monoolein, 1,2-diolein, 1,3-diolein, and triolein were acquired from Sigma-Aldrich. The mobile phases of HPLC were chromatographically pure acetonitrile and dichloromethane. The rest of the chemicals and reagents were of analytical grade and commercially acquired.

### 2.2. Assaying of Enzyme Activity

The enzyme's esterification activity was determined by the model reaction of oleic acid esterification by glycerol. In the usual experiment, the following reagents were combined and incubated at 50°C at 200 rpm in a shaking 50-mL Erlenmeyer flask topped with a septum: 8.9 mmol oleic acid, 3.0 mmol glycerol, 1.6 g 4 Å molecular sieves, and the enzyme. One unit was quantified as the portion of enzyme that catalyzed the esterification of 1 mmol oleic acid with glycerol per minute, and the specific activity of the enzyme was 34.5 U/g.

### 2.3. Procedure for Preparation of 1,3-Diolein

The* Candida *sp. 99–125 lipase-mediated esterification of oleic acid by glycerol was carried out in a 50-mL flask on a rotary shaker at 200 rpm at 50°C. The makeup of the reaction was 1.52 mmol oleic acid, 0.60 mmol glycerol, 10 mL solvent, and 1.52 U* Candida *sp. 99–125 lipase. 4 Å molecular sieves (270 mg) were added to adsorb water. Specimens (50 *μ*L) were obtained and centrifuged to obtain the supernatant for subsequent HPLC and GC analysis.

### 2.4. Analysis of the Samples

1-Monoolein, 2-monoolein, 1,2-diolein, 1,3-diolein, and triolein contents in the reaction combination were examined according to the method of Duan et al. [8]. Shimadzu 20A HPLC with ELSD (evaporative light scattering detector) was used. External standards (1-monoolein, 2-monoolein, 1,2-diolein, 1,3-diolein, and triolein) were used to prepare eight concentrations of calibration solutions. A 2-*μ*L sample and 1 mL acetone were fully combined. The chromatographic column was a C18 column (5 *μ*m, 250 mm×4.6 mm) (Dikma Technology, PLATISIL ODS, China). A gradient elution with acetonitrile-acetic acid (99.85/0.15)/dichloromethane was used for the reaction mixture analysis (0-4.0 min, 100/0; 4.0-12.0 min, 90/10; 25.0-30.0 min, 70/30; 35.0-45.0 min, 20/80; 55.0-60.0 min, 100/0). The flow rate was controlled at 1.5 mL/min. The column temperature and drift pipe temperature were 40°C and 70°C, respectively, and the nitrogen pressure was 320 kPa. The retention times were 3.75 min, 4.54 min, 23.12 min, 23.90 min, and 42.91 min for 2-monoolein, 1-monoolein, 1,3-diolein, 1,2-diolein, and triolein, respectively. Oleic acid and glycerol were established via the procedures documented by Du et al. [15]. All of the documented data are averages of experiments conducted at minimum in duplicate.

### 2.5. Identification of Rate Constants

**Scheme 1:**
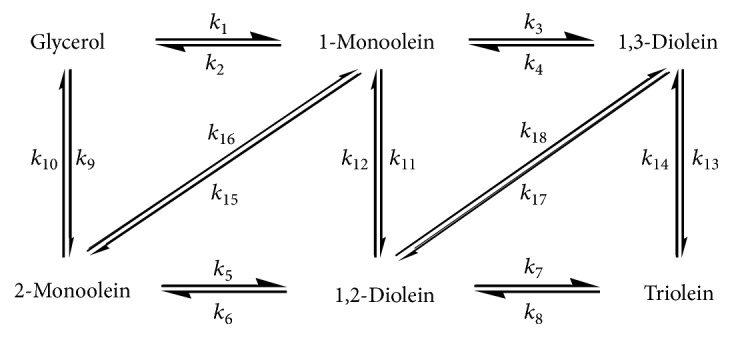
The lipase-mediated esterification of oleic acid with glycerol.

To investigate the kinetic behaviors in* Candida *sp. 99-125 lipase-mediated process of preparing 1,3-diolein in tested solvents, the synthetic process is depicted in Scheme 1. The reaction rates follow second-order kinetics except the acyl migration rates (follow first-order kinetics) [3, 16]; the mass transfer limitation is neglected. According to these assumptions, the differential equations are documented as follows, and OA, 1-MO, 2-MO, 1,3-DO, 1,2-DO, TO, and Gly refer to oleic acid, l-monoolein, 2-monoolein, 1,3-diolein, 1,2-diolein, triolein, and glycerol in the equations, respectively.

(1) dOAdt=k21−MO+k102−MO+k41,3−DO+k6+k121,2−DO+k8+k14TO−k1+k9Gly+k3+k111−MO+k52−MO+k71,2−DO+k131,3−DO·OAd1−MOdt=k1OAGly+k41,3−DO+k121,2−DO+k162−MO−k2+k5+k3+k11OA1−MOd2−MOdt=k61,2−DO+k9OAGly+k151−MO+k5OA+k10+k162−MOd1,3−DOdt=k31−MOOA+k14TO+k181,2−DO−k4+k13OA+k171,3−DOd1,2−DOdt=k52−MO+k111−MOOA+k8TO+k171,3−DO−k6+k7OA+k12+k181,2−DOdTOdt=k71,2−DO+k131,3−DOOA−k8+k14TOdGlydt=k21−MO+k102−MO−k1+k9·OAGly

The rate constants were acquired by solving the equations above utilizing the adaptive step-size Runge–Kutta strategy in the nonlinear regression method by the Levenberg–Marquardt algorithm, to obtain the best fit among the experimental data and the results determined [3, 16]. The rate constants of concern for the positional selectivity of* Candida *sp. 99–125 lipase are shown in Table 1.

### 2.6. Lipase-Mediated Larger-Scale 1,3-Diolein Synthesis

The enzymatic esterification was conducted in a 50-mL Erlenmeyer flask topped with a septum on a rotary shaker at 200 rpm. The reaction mixtures included the following: oleic acid (fixed at 35.4 mmol), glycerol,* t*-butanol, and* Candida *sp. 99–125 lipase. 4 Å molecular sieves were utilized for adsorbing the byproduct water, and the amount added was 10 times the predicted water quantity. Fifty-microliter samples were removed, and they were centrifuged to obtain the supernatant for subsequent HPLC and GC analysis. The routine single-factor experiments were applied for the process optimization.

### 2.7. Operational Stability of Candida sp. 99–125 Lipase

The reactions were performed under optimized conditions in larger-scale experiments (35.4 mmol oleic acid, 11.8 mmol glycerol, 34.5 U* Candida *sp. 99–125 lipase, 6.0 mL* t*-butanol, 6.4 g 4 Å molecular sieves, 50°C, and 7 h) acquired by the typical single-factor experiments in a 50-mL flask on a rotary shaker at 200 rpm. When the reaction was completed, the lipase powders and the molecular sieves were divided from the reaction via filtration with Whatman #1 filter paper and then rinsed with* t*-butanol in a Buchner funnel minus filter paper. The lipase powders and* t*-butanol could pass the holes of Buchner funnel, while the molecular sieves not. Thus, the molecular sieves were isolated from the lipase powders which can be separated with* t*-butanol by filtration using Whatman #1 filter paper and then directly used during the further batch.

### 2.8. The Kg Level Experiments

The enzymatic esterification proceeded in a 5.0 L stirred tank reactor at 200 rpm. The reaction combinations are as follows: 3.54 mol oleic acid, 1.18 mol glycerol, 0.6 L* t*-butanol, and 3450 U* Candida *sp. 99–125 lipase. 4 Å molecular sieves were included to adsorb the byproduct water. The 5-mL samples were removed and centrifuged to obtain the supernatant for evaluation.

### 2.9. Purification of 1,3-Diolein

After the reactions, the enzyme powders and the molecular sieves were separated by filtration, and then the reaction mixtures were transferred to a rotary evaporator (BUCHI, R-215, Switzerland), in which the reaction media* t*-butanol could be removed at 40°C when the vacuum was 130 mbar. The purification of 1,3-diolein was performed on a molecular distillation (UIC GmbH, KDL5, Germany) at 180°C and 0.1 mbar.

## 3. Results and Discussion

It is well known that the enzyme performance depends very much on the type of the reaction medium, for example, widely different activity and selectivity are observed with different nature of the organic solvent [18, 19]. With the goal of preparing 1,3-diolein, the solvents with log⁡*P* values from -0.23 to 4.5 acted as the reaction media for the esterification of oleic acid with glycerol catalyzed by* Candida *sp. 99–125 lipase. Diolein yields are provided in Table 1 (column 5). As one can see, the diolein yield obviously relied on the polarity characteristics of the reaction media. The lowest diolein yield (56.5%±1.5%) occurred in acetone with the smallest log⁡*P* value (-0.23). Moderate values were acquired in the comparably hydrophilic solvents (Table 1, Entries 2-5). Greater diolein yields were attained in the hydrophobic solvents (Table 1, Entries 6-11). When the reaction media were* n*-hexane,* n*-heptane, and* n*-octane, greater than 86% yield was achieved. In addition, reaction times were noticeably different in each of the solvents. The reaction time was lowered with the increasing log⁡*P* values, which suggested that enzyme activity was greater in the higher log⁡*P* solvent. The higher enzyme activity involved in this case might be attributable to the fact that the solvents with greater log⁡*P* could retain the microenvironment moisture surrounding the active area of* Candida *sp. 99–125 lipase to a greater extent, resulting in a more stable conformation of the enzyme, thereby eluding the deactivation that occurs as a result of the lack of essential water [20, 21].

In contrast, the 1,3-diolein to 1,2-diolein ratio shown in Table 1 (column 6) was significantly impacted by the reaction media. The largest ratio of 1,3-diolein to 1,2-diolein (28.3) was documented in the most hydrophilic solvent (acetone). The 1,3-diolein to 1,2-diolein ratio was lowered quickly with the elevating log⁡*P* value. The lowest 1,3-diolein to 1,2-diolein ratio was 5.2, which was observed in* n*-octane (the most hydrophobic solvent). Specifically, the 1,3-diolein to 1,2-diolein ratio was influenced substantially in the tested hydrophobic solvents (Table 1, Entries 6-11), which impacted diolein yield to a minor extent. As is recognized, the ratio of 1,3-diolein to 1,2-diolein relied on the favorable selectivity of* Candida *sp. 99–125 lipase for the* sn-1* hydroxyl over the* sn-2* hydroxyl of glycerol and the migration of the acyl group (which can be neglected due to the minor impact; data not shown).

According to the above results, a detailed evaluation of the positional selectivity of* Candida *sp. 99–125 lipase based on medium engineering was explored. From Table 1 (columns 7–10), it can be viewed that* k*_*1*_,* k*_*3*_,* k*_*9,*_ and* k*_*11*_ were improved with the increasing log⁡*P* values. It substantially improved the diolein yield that occurred from the rise of log⁡*P* value.* k*_*1*_ was larger than* k*_*9*_ in each of the tested solvents, demonstrating that the particular selectivity of* Candida *sp. 99–125 lipase towards the* sn-1* hydroxyl of glycerol was far greater than the one to the* sn-2* hydroxyl. However, the increase times of* k*_*1*_ were substantially less than those of* k*_*9*_ with increasing log⁡*P* values. Therefore,* k*_*1*_/*k*_*9*_ was lowered obviously (from 14.16 to 1.71). It was shown that the favorable selectivity of* Candida *sp. 99–125 lipase towards* sn-1* hydroxyl over* sn-2* hydroxyl became lessened with the increase in solvent log⁡*P* values. When the reaction media were* n*-hexane,* n*-heptane, and* n*-octane, the values of* k*_*1*_/*k*_*9*_ were 2.27, 1.83, and 1.71, respectively. As the amount of* sn-1* hydroxyl was two times the* sn-2* hydroxyl in glycerol, if the value of* k*_*1*_/*k*_*9*_ was near 2.00, it could be seen that the particular selectivity of* Candida *sp. 99–125 lipase towards* sn-1* hydroxyl and* sn-2* hydroxyl was comparable. It is beneficial for generating 2-monoolein and 1,2-diolein utilized as emulsifiers or surfactants in the food industry [22, 23].

In addition, while the* k*_*3*_ increase times were smaller than those of* k*_*11*_ with the rise of log⁡*P* values,* k*_*3*_ was still noticeably larger than the correlated* k*_*11*_, so that* k*_*11*_ could be ignored. It was demonstrated that the particular selectivity of* Candida *sp. 99–125 lipase towards the* sn-1* hydroxyl of 1-monoolein was noticeably greater than the one towards the* sn-2* hydroxyl in each of the tested solvents. Further,* k*_*3*_/*k*_*11*_ was larger than* k*_*1*_/*k*_*9*_ in all of the solvents, indicating that the greater preferential selectivity of* Candida *sp. 99–125 lipase to the* sn-1* hydroxyl over the* sn-2* hydroxyl occurred in the molecule of 1-monoolein.

Based on the attained outcomes mentioned above, the diolein yield was higher, while the preferential selectivity of* Candida* sp. 99–125 lipase to the* sn-1* hydroxyl over the* sn-2* hydroxyl of glycerol was lessened, leading to a decreased 1,3-diolein to 1,2-diolein ratio in the solvent with greater log⁡*P* values. Thus, the relatively hydrophilic solvent of* t*-butanol was chosen as the best medium for* Candida *sp. 99–125 lipase-mediated 1,3-diolein preparation.

**Figure 1:**
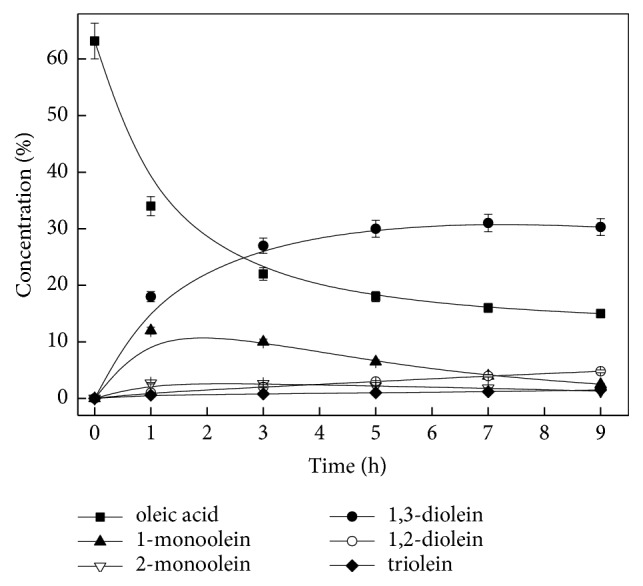
The time course of the esterification of oleic acid with glycerol for 1,3-diolein synthesis catalyzed by* Candida *sp. 99-125 lipase. Reaction conditions: 35.4 mmol oleic acid, 11.8 mmol glycerol, 34.5 U lipase, 6.0 mL* t*-butanol, and 6.4 g 4 Å molecular sieves, 50°C.

To additionally evaluate the possibility of using* Candida *sp. 99–125 lipase for the industrial production of 1,3-diolein, studies on the larger-scale experiments, operational stability of lipase, and 1,3-diolein purification were developed. The impact of some substantial reaction factors including the reaction temperature, amount of lipase, the oleic acid to glycerol molar ratio, and the amount of* t*-butanol were examined systematically by routine single-factor experiments (data not shown). The outcomes revealed that diolein yield and the 1,3-diolein to 1,2-diolein ratio attained 75.3% and 7.8, respectively, with the optimal reaction conditions (35.4 mmol oleic acid, 11.8 mmol glycerol, 34.5 U* Candida* sp. 99–125 lipase, 6.0 mL* t*-butanol, and 6.4 g 4 Å molecular sieves, 50°C, 7 h). Under reaction conditions that were optimal, the process curves of* Candida* sp. 99–125 lipase-mediated esterification method to prepare 1,3-diolein are shown in Figure 1. Besides the substrate concentration (oleic acid) and the target product (1,3-diolein), the concentration of the intermediates (1-monoolein) and the by-products (2-monoolein, 1,2-diolein, and triolein) are also revealed. As can be observed, the biggest concentration of 1,3-diolein was achieved at 7 h.

**Figure 2:**
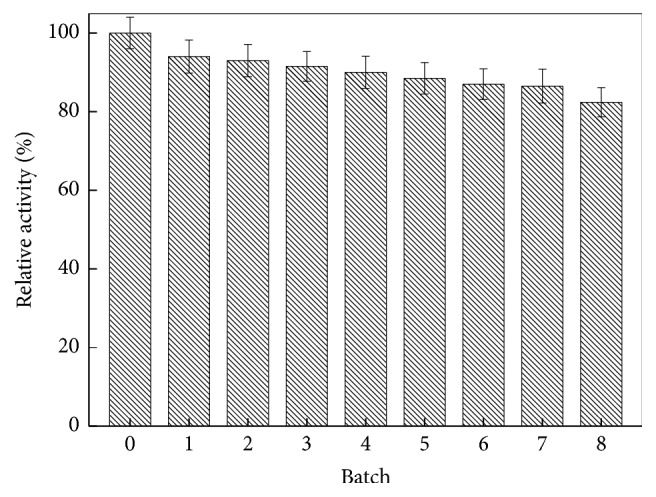
The operational stability of* Candida *sp. 99-125 lipase. Reaction conditions: 35.4 mmol oleic acid, 11.8 mmol glycerol, 34.5 U lipase, 6.0 mL* t*-butanol, and 6.4 g 4 Å molecular sieves, 50°C, 7 h.

Following the reaction,* Candida* sp. 99–125 lipase was divided via filtration and then used in the following batch with the goal of understanding the enzyme's operational stability.  Figure 2 shows enzyme activity which was characterized by the 1,3-diolein concentration at the end of the reaction as a function of the reaction batch, in which batch 0 is the 1,3-diolein concentration catalyzed by fresh lipase in the initial trial, which was determined to be 100%. One can see that 82.4% of the initial activity remained after eight batches, suggesting that this lipase is cost-effective.

Furthermore, the product was purified by the rotary evaporation for solvent removing followed by the molecular distillation for the removal of the excess oleic acid and by-products.* t*-Butanol is a commercial solvent obtained from isobutylene and has emerged as a stable medium for drugs and spices synthesis. It is a relatively nontoxic compound with acute effects similar to ethanol and has high vapor pressure (31 mm Hg at 20°C) and low boiling point (82.8°C) for easy recovery. In this experiment,* t*-butanol separated by the rotary evaporation could be recycled for the synthetic reaction. In addition, in Table 2, one can view that, following purification, the products' diolein content may reach 95.1%; meanwhile, the 1,3-diolein to 1,2-diolein ratio was 7.1, which was a bit less than that prior to purification (7.8). The possible reason may be that the acyl migration happened during the purification process especially in the molecular distillation which performed at a higher temperature.

When the kilogram-level experiments were conducted in a 5.0 L stirred tank reactor at 200 rpm using the enlarged reaction conditions, the diolein yield and the 1,3-diolein to 1,2-diolein ratio were 70.7% and 7.5, respectively. These slightly lower achievements may be resulted from the decreasing mass transfer efficiency between enzyme particles and substrates during a larger-scale process. Besides, based on the purification procedure, the diolein content and the 1,3-diolein to 1,2-diolein ratio in the products could reach 94.3% and 7.2, respectively. This undoubtedly confirmed the practicability of the enzymatic process and the efficiency of the purification strategy for the product.

## 4. Conclusions

To summarize, this research clearly showed that the log⁡*P* values of the solvents displayed noticeable impact on the positional selectivity of* Candida *sp. 99–125 lipase in 1,3-diolein synthesis by glycerol esterification of oleic acid. It is speculated that the reaction solvents with suitable polarity characteristics contributed much more significantly to* Candida *sp. 99–125 lipase behaviors, thus affording the promising 1,3-diolein yield. Moreover, the encouraging lipase-mediated larger-scale experiments and stability investigation suggest the possibility of using* Candida *sp. 99–125 lipase for the 1,3-diacylglycerol industrial synthesis.

## Figures and Tables

**Table 1:** Solvent effect on the enzymatic 1,3-diolein synthesis.

Entries	Solvent	log⁡*P* [17]	Time (h)	Diolein yield (%)	1,3-Diolein/1,2-diolein	*k*_*1*_	*k*_*3*_	*k*_*9*_	*k*_*11*_	*k*_*1*_/*k*_*9*_	*k*_*3*_/*k*_*11*_
1	Acetone	-0.23	10	56.5±1.5	28.3	0.524	0.639	0.037	4E-5	14.16	15975
2	Tetrahydrofuran	0.49	8	70.7±1.8	24.1	0.908	0.931	0.089	7E-4	10.20	1330
3	*t*-Butanol	0.8	7	78.2±2.5	20.2	1.012	1.167	0.113	0.0028	8.96	416.8
4	4-Methyl-2-pentanone	1.31	6	80.4±2.2	16.1	1.038	1.272	0.131	0.0075	7.92	169.6
5	Chloroform	2.0	6	82.6±2.6	10.7	1.104	1.305	0.146	0.014	7.56	93.2
6	Toluene	2.5	5	84.3±2.3	7.8	1.163	1.341	0.204	0.021	5.70	63.9
7	Tetrachloromethane	3.0	5	85.4±2.9	6.9	1.262	1.521	0.289	0.030	4.37	50.7
8	Cyclohexane	3.2	5	85.7±2.1	5.9	1.341	1.818	0.476	0.042	2.82	43.3
9	*n*-Hexane	3.5	5	86.2±2.4	5.8	1.414	1.896	0.622	0.045	2.27	42.1
10	*n*-Heptane	4.0	5	86.5±2.5	5.3	1.504	1.913	0.821	0.046	1.83	41. 6
11	*n*-Octane	4.5	5	86.7±2.3	5.2	1.521	2.125	0.892	0.057	1.71	37.3

**Table 2:** The contents of various components during the purification process (%).

	*t*-Butanol	Oleic acid	1-Monoolein	2-Monoolein	1,3-Diolein	1,2-Diolein	Triolein
Before solvent removal	29.94	19.12	5.96	2.13	31.02	3.98	1.55
After solvent removal	-	29.97	9.22	3.47	48.47	6.42	2.44
After molecular distillation	-	-	-	-	83.33	11.81	4.87

## Data Availability

The data used to support the findings of this study are included within the article.
